# Isotopically enhanced triple-quantum-dot qubit

**DOI:** 10.1126/sciadv.1500214

**Published:** 2015-05-29

**Authors:** Kevin Eng, Thaddeus D. Ladd, Aaron Smith, Matthew G. Borselli, Andrey A. Kiselev, Bryan H. Fong, Kevin S. Holabird, Thomas M. Hazard, Biqin Huang, Peter W. Deelman, Ivan Milosavljevic, Adele E. Schmitz, Richard S. Ross, Mark F. Gyure, Andrew T. Hunter

**Affiliations:** HRL Laboratories, LLC, 3011 Malibu Canyon Road, Malibu, CA 90265, USA.

**Keywords:** Qubits, quantum dots, isotopically enhanced semiconductors, semiconductor heterostructures, quantum control, quantum information

## Abstract

Like modern microprocessors today, future processors of quantum information may be implemented using all-electrical control of silicon-based devices. A semiconductor spin qubit may be controlled without the use of magnetic fields by using three electrons in three tunnel-coupled quantum dots. Triple dots have previously been implemented in GaAs, but this material suffers from intrinsic nuclear magnetic noise. Reduction of this noise is possible by fabricating devices using isotopically purified silicon. We demonstrate universal coherent control of a triple-quantum-dot qubit implemented in an isotopically enhanced Si/SiGe heterostructure. Composite pulses are used to implement spin-echo type sequences, and differential charge sensing enables single-shot state readout. These experiments demonstrate sufficient control with sufficiently low noise to enable the long pulse sequences required for exchange-only two-qubit logic and randomized benchmarking.

## Teaser

Three coupled quantum dots in isotopically purified silicon enable all-electrical qubit control with long coherence time.

## INTRODUCTION

A decade ago, electrically controlled double quantum dots were experimentally demonstrated as a possible platform for semiconductor-based quantum information processing (*1*). A major difficulty recognized at the time was rapid decoherence from inhomogeneous magnetic noise due to nuclear spins intrinsic to the GaAs semiconductor host. This suggested that the nuclei would have to be either better controlled or eliminated. Rapid, single-shot measurement in GaAs quantum dots (*2*, *3*) has recently enabled measurement of the random nuclear magnetic field on a time scale faster than its diffusion time. These measurements allowed improved use of nuclear spins for control, boosting control fidelity and leading to the observation of quantum coherence times of tens of microseconds in GaAs (*4*, *5*).

Even longer quantum coherence times are available if nuclear spins are altogether removed, which is possible in silicon-based systems. Electron spin resonance measurements of ensembles of donor-bound spins in isotopically purified ^28^Si material have shown coherence times approaching seconds (*6*, *7*). Recent results using electron spin states in isotopically natural silicon-based quantum dots and single impurities (*8*–*12*) show substantial reductions of nuclear magnetic noise compared to GaAs. These improvements are even more dramatic in demonstrations controlling single quantum dots or single impurities using microwaves in isotopically enhanced material, in which coherence times comparable to bulk results are observed (*13*, *14*). The proximal metal gates, oxides, and material interfaces required in those experiments do not drastically impair quantum coherence.

Despite these promising improvements in spin coherence times, all-electrical universal control of spin qubits in silicon, using any isotopic content, remains an outstanding goal. The importance of all-electrical control relates to the ability to control multiple devices in a single chip because it does not require static magnetic field gradients [for example, from micromagnets, as in refs. (*12*, *15*)] or microwaves for electron spin resonance [as in refs. (*9*, *11*, *14*)], which are challenging to isolate to individual devices. All-electrical control of semiconductor spin qubits is possible using spins in triple quantum dots coupled via the exchange interaction (*16*). Triple dots controlled this way have only recently been fabricated and tested in the noisier GaAs system (*17*, *18*).

In this report, we demonstrate universal quantum control of a triple-quantum-dot qubit in an isotopically enhanced silicon system, enabling all-electrical operation with drastically reduced nuclear magnetic noise. The correspondingly improved quantum coherence is measured using a fast, single-shot spin readout mechanism. With magnetic noise reduced, charge noise becomes the dominant limitation to control fidelity. To measure charge noise dynamics, we utilize the universal control of a triple dot to construct a composite pulse sequence that refocuses exchange noise using exchange pulses, and the resulting echo data fit well to a 1/*f* noise model.

## RESULTS

**Fig. 1:**
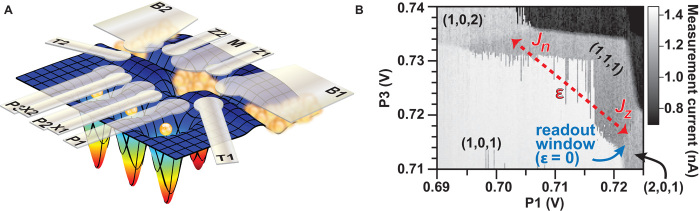
Triple-quantum-dot device. (**A**) Schematic diagram of a triple-dot device depicting the gate layout and the resulting electrostatic control of the potential landscape. Electrons are schematically depicted as yellow spheres. The lateral triple dot is formed underneath gates labeled P1, P2, and P3. Gates X1 and X2 affect the tunnel coupling (exchange) between dots P1 and P2 and dots P2 and P3, respectively. A local charge-sensing quantum dot is formed under the gate labeled M, whose tunnel rates to the bath are controlled by gates Z1 and Z2. The source of electrons for the system is provided by the two-dimensional electron gas (2DEG) formed under bath gates B1 and B2. Initialization and loading/unloading electrons from the outer dots (P1 and P3) to the 2DEG are controlled by the gates labeled T1 and T2. (**B**) Charge stability diagram of a triple dot in the (1,1,1) [P1,P2,P3] configuration plotted as a function of the DC bias applied to P3 versus P1. The grayscale in the plot is the current measured from the charge sensor created underneath the M gate shown in (A). The charge state of (1,0,1) bleeding into the (1,1,1) state occurs owing to the slow loading rate of the first electron in the middle dot (P2) relative to the scan rate of the plot. The red dotted line shows the P1 and P3 voltages used to define the detuning bias ε.

The triple quantum dot studied in this work is fabricated by using multiple layers of patterned gates deposited above an undoped Si/SiGe heterostructure. This design addresses various outstanding challenges for Si-based quantum dot development unrelated to nuclear magnetism. Principal among these are the larger effective mass of conduction electrons in silicon in comparison to GaAs and, subsequently, an increased sensitivity to electrostatic potential fluctuations caused by sample impurities and defects. The sample uses only accumulation gates that draw electrons from ohmic contacts under the target dot areas (*14*, *19*–*21*) into a strained ^28^Si enriched quantum well below. The residual ^29^Si content of this layer is 800 ppm, comparable to devices in refs. (*14*, *22*). (Secondary ion mass spectroscopy studies of similarly grown devices confirm this ^29^Si content within the quantum well layer.) Figure 1A depicts a schematic diagram of the patterned gates used to create the triple dot, as well as the integrated quantum dot charge sensor that forms underneath the gate labeled “M.” Fabrication and operation are similar to previous work (*21*), except that this triple-dot device has additional gates to accommodate the occupancy of the third dot (gates labeled P3 and X2).

The number of electrons in each of the three quantum dots is inferred from changes in the current through the quantum dot charge sensor, enabling the tuning of the triple dot into the (P1,P2,P3) = (1,1,1) charge configuration. Figure 1B shows the charge stability diagram of the (1,1,1) state by varying the DC biases on the P1 and P3 gates while holding the other gates fixed (except that the M-gate voltage is changed as other voltages are swept to maintain constant charge sensitivity). The qubit manipulations presented below are performed while maintaining the (1,1,1) charge state, and the detuning voltage, ε, is a linear function of the pulse amplitudes applied only to gates P1 and P3. The state of the (1,1,1) qubit is measured using spin-to-charge conversion based on Pauli spin blockade. Here, the middle electron is pulsed past the boundary between (1,1,1) and (2,0,1) where the singlet and triplet spin states of the (2,0,1) charge configuration are separated by an energy splitting *E*_ST_. The singlet state is able to freely transfer into (2,0,1), whereas transport of the triplet state is blocked via the Pauli exclusion principle, allowing one to infer the state of the qubit via charge sensing (*1*, *4*, *8*). For the present device, readout at ε = 0 and initialization is performed only in the (2,0,1) configuration where the singlet-triplet splitting *E*_ST_ was observed to be about 150 μeV. (The singlet-triplet splitting for the (1,1,1)-(1,0,2) charge transition was too small to be observed, presumably due to a small valley splitting on the corresponding side of the device.)

**Fig. 2:**
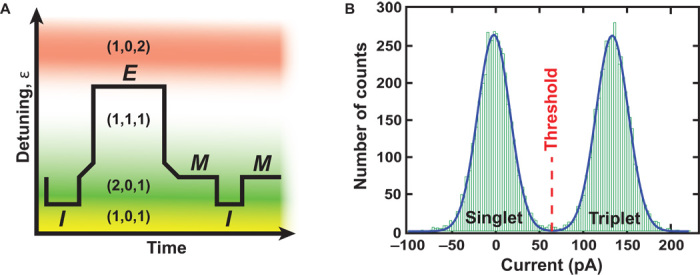
Singlet-triplet measurement. (**A**) Schematic diagram of the pulse sequence used in double-dot $T_{2m}^{*}$ measurements. Different charge regions for the three dots are labeled by color and number. Singlet initialization (*I*) occurs via tunneling of a bath electron, transitioning from (1,0,1) to (2,0,1). This is followed by a ramp to the (1,1,1) state for evolution (*E*). The measurement sequence is composed of two charge-sensing segments (*M*) with singlet initialization inserted between them. The second segment measures the current corresponding to a singlet. A differential measurement is achieved by taking the difference in average current between the two segments. (**B**) Histogram of 10,000 repeated single-shot measurements implementing the pulse sequence in (A) with a 20-μs evolution in (1,1,1). The distributions of singlet and triplet currents are both Gaussian with equal root-mean-square widths of 18.55 pA, representing the measurement noise. The vertical dashed red line (*I* = 64.2 pA) represents the threshold current used to discretize the signal.

Discrimination between charge states (2,0,1) and (1,1,1) is measured by an 18% change in the electric current through the quantum dot charge sensor. Digitization of the current at a rate much faster than the measurement time allows us to implement a differential measurement technique in software that is robust to low-frequency current noise. The technique involves taking the difference in average current between two measurement segments: the first captures either triplet or singlet depending on the qubit evolution, and the second is performed after a singlet initialization, thus measuring the singlet current only. Figure 2A depicts a representative pulse sequence applied to the P1 and P3 gates. Figure 2B shows a histogram of 10,000 consecutive single-shot measurements depicting a bimodal distribution in which the mean differential current for singlet [(2,0,1) charge state] is *I* = −3.4 pA and the mean differential triplet current is *I* = 131.8 pA. More details of this measurement technique appear in the Materials and Methods section. Because each measurement instance is complete in a time substantially lower than *T*_1_, measured to be greater than 100 ms near zero applied field, this amounts to a single-shot measurement, as previously demonstrated in both GaAs and silicon devices (*2*, *3*, *23*, *24*).

Magnetic gradient noise in our device is expected to be reduced owing to the use of isotopically purified ^28^Si. This is more easily studied in the context of the singlet-triplet qubit subspace of a double quantum dot for which, with a global magnetic field large enough to suppress hyperfine flip-flops, magnetic field gradients act as *x* rotations and exchange pulses act as orthogonal *z* rotations (*1*, *4*, *8*, *25*). This subspace allows for single-pulse spin-echo experiments. [In the triple-dot case, measurements to refocus magnetic noise are more complicated (*26*, *27*).]. Therefore, to perform noise magnetometry, we tune our triple-dot system as a double dot. We decouple the third dot by reducing the bias on gate X2, which isolates the electron under P3 from the other two.

**Fig. 3:**
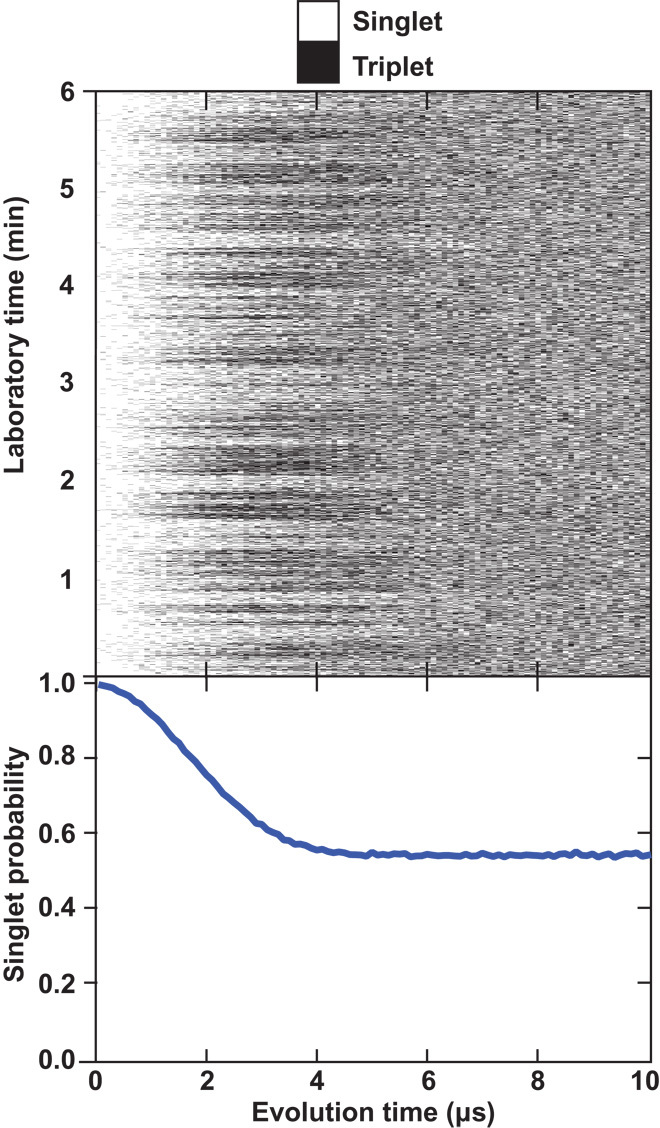
Double-dot $T_{2m}^{*}$. Each pixel of the top plot represents a single-shot measurement of singlet or triplet as a function of evolution time (horizontal axis) using the pulse sequence shown in Fig. 2A in the *J*_*z*_ = 0 double-dot (1,1) configuration. Each collection of experiments is repeated as a function of laboratory time (vertical axis). The bottom plot shows the ensemble average of singlet probability where each evolution time is averaged 20,000 times over about 33 min. A Gaussian fit of the form exp[−(*t*/$T_{2m}^{*}$)^2^] gives a $T_{2m}^{*}$ value of 2.31 ± 0.01 μs.

As previously observed in double dots (*1*, *8*), magnetic noise manifests as oscillations between singlet states and triplet states caused by the spin flip-flop energy splitting Δ = *g*μ_B_(*B*_2_ − *B*_1_), where *g ≈* 2.0 is the gyromagnetic ratio in silicon, μ_B_ is the Bohr magneton, and *B*_*j*_ is the magnetic field in dot *j* due to all sources including effective hyperfine fields. The average over an ensemble of single-shot measurements results in a Gaussian decay, exp[−〈Δ^2^〉*t*^2^/2ℏ^2^], and we define $T_{2m}^{*}=\hbar \sqrt{\frac{2}{\langle \Delta^{2}\rangle }}$. The “m” subscript reminds us that this dephasing time refers to magnetic noise, as opposed to charge noise. At zero applied field, the ensemble-averaged data (over a time span of 33 min) in Fig. 3 show a $T_{2m}^{*}$ of 2.31 ± 0.01 μs. This value of $T_{2m}^{*}$ is substantially longer than that seen in comparable experiments in GaAs and isotopically natural Si/SiGe quantum dots, where observed values are 10 ns (*1*) and 360 to 900 ns (*8*, *12*) respectively. This demonstrates that isotopic purification successfully improves the dephasing time.

A critical question, however, is whether the $T_{2m}^{*}$ we observe is due to the remaining 800 ppm ^29^Si nuclear isotopes. Initial evidence of nonnuclear effects comes from the observation that Δ has a mean value that increases linearly with applied magnetic field, suggesting paramagnetism far greater than expected for nuclei. Furthermore, time-dependent fluctuations of Δ, directly observable with our single-shot measurements, show that this variable fluctuates with a magnetic-field–dependent power spectral density uncharacteristic of diffusing nuclear magnetization. Spin-echo measurements show Hahn-echo *T*_2m_ times increasing from 7 to 700 μs with increasing applied magnetic field, corroborating direct time-domain measurements. These *T*_2m_ values are substantially less than other spin-echo measurements in 800 ppm material (*6*, *14*); more notably, these measurements show that gradient fluctuations change their correlation time most dramatically at magnetic field values suggestive of electronic magnetism. Details of these measurements may be found in the Supplementary Materials. Although the origin of this noise is unknown, it far exceeds numerical estimates for possible sources of paramagnetic gradients originating from the bulk Si/SiGe material, suggesting that the source is inadvertently introduced to the sample during the gate fabrication process. Regardless of the source, this magnetic noise could be dynamically decoupled (*26*) with sufficiently high-quality exchange pulses.

**Fig. 4:**
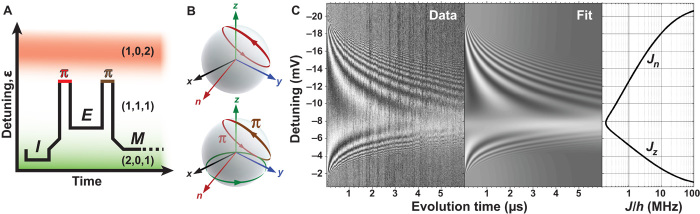
Triple-dot Rabi-Ramsey. (**A**) Schematic diagram of the composite pulse sequence applied to enable measurements of both *J*_*z*_ and *J*_*n*_ in (1,1,1). The pulse sequence is as follows: initialization (*I*) of a singlet in (2,0,1), a π-pulse about the *n* axis, an evolution segment (*E*) that varies in both time and detuning, another π-pulse about the *n* axis, and then a measurement (*M*). Although not shown, the same differential measurement technique as in Fig. 2A is used. (**B**) Corresponding paths on triple-dot Bloch spheres for different *J*_*z*_ and *J*_*n*_ regimes. Top: For very negative detunings, both π-pulses and free evolution drive rotations about the *n* axis (red circle). Bottom: For less negative detunings, the π-pulses drive the free evolution into a southern line of latitude to allow measurement of rotations about the *z* axis (green circle). (**C**) Measured (Data) and theoretical (Fit) Rabi and Ramsey oscillations due to *J*_*n*_ and *J*_*z*_ rotations in the (1,1,1) state plotted as a function of detuning versus evolution time using the pulse sequence depicted in (A). The grayscale shows the average over 50 single-shot measurements, with white (black) corresponding to singlet (triplet). The rightmost panel shows *J*_*n*_ and *J*_*z*_ exchange rates as extracted from the fit.

To demonstrate universal control of a qubit and examine the quality of our exchange pulses, we measure triple-dot Rabi and Ramsey fringes. We couple the third electron by using a bias on gate X2 that is increased relative to the bias used in the $T_{2m}^{*}$ experiments. The addition of the third dot introduces a second distinct axis of exchange-based control, improving the visibility of Rabi fringes in comparison to double-dot Rabi experiments (*1*, *8*), which rely on adiabatic passage into random hyperfine states. In a triple-dot qubit, state |0〉 is initialized by preparing a spin singlet in the first two dots via tunneling into the ground state of the (2,0,1) charge configuration. Exchange between the first two dots at rate *J*_*z*_ generates rotations around the *z* axis of the Bloch sphere, on which |0〉 is the north pole. Exchange between dots 2 and 3 at rate *J*_*n*_, which occurs when biasing toward the (1,0,2) charge regime, generates rotations around the axis $\hat{n}$ *= $\hat{z}$* cosϕ + $\hat{x}$ sinϕ, where ϕ = 120° (*16*, *18*, *27*). With this basis established, the pulse sequence used to observe triple-dot exchange oscillations is shown in Fig. 4, A and B. Exchange oscillations about both the *n* and *z* axes are observable if they are preceded and followed by a π-pulse about the *n* axis. For ε near (1,0,2), we see Rabi oscillations corresponding to rotations only about the *n* axis; these are the upper oscillations in Fig. 4C. As our detuning bias approaches the center of the (1,1,1) region, these oscillations become slower and eventually disappear. As we approach (2,0,1), more oscillations appear. These are Ramsey fringes, corresponding to a fixed initial π rotation about the *n* axis, a variable-time rotation about the *z* axis, and another fixed π rotation about the *n* axis.

These oscillations, as well as similar oscillations observed in GaAs triple dots (*18*), show damping due to a combination of charge noise and magnetic gradient noise. The data of Fig. 4C fit well to a model based on the analysis in ref. (*27*). Details of this model appear in the Supplementary Materials. The fit indicates magnetic-noise–induced decay, which may be modeled as though each dot had a Gaussian distribution of random Zeeman frequencies with width σ_B_*/h* = 63 ± 5 kHz, which would correspond to a double-dot $T_{2m}^{*}$ = *ħ*/σ_B_ of 2.5 ± 0.2 μs, consistent with Fig. 3. Charge noise damping fits well to a Gaussian distribution of noisy exchange values *J*_*k*_ (for *k* = *z* or *n*) with σ_*J*_ = |d*J*_*k*_*/*dε|σ_ε_, corresponding to charge noise–induced detuning-dependent dephasing rate $T_{2e}^{*}=\sqrt{2}/\sigma_{J}$. Here, our fit reveals an effective detuning voltage linewidth σ_ε_ = 70 ± 8 μV. A critical question, not evident from the Rabi damping alone, is whether this damping arises from quasistatic drift or faster noise processes. Such a question is addressed via spin-echo–like electrometry techniques.

In general, spin-echo–like refocusing occurs when rotations about one axis are interrupted by a π rotation about an orthogonal axis. To completely refocus noisy exchange oscillations, a control axis orthogonal to exchange rotations is required. In GaAs double dots, hyperfine field gradients have been used for this task (*28*). In a triple-dot exchange-only qubit, however, the two fundamental control axes are nonorthogonal. To enable exchange refocusing and subsequent electrometry, we construct a composite sequence to enable a refocusing π rotation around the *y* axis; we refer to this composite rotation as a Y-pulse. This is chosen because the *y* axis is orthogonal to both *n* and *z* axes of the Bloch sphere, enabling the refocusing of exchange noise on either axis. We refer to the sequence in which exchange oscillations are refocused by a Y-pulse as Y-echo.

**Fig. 5:**
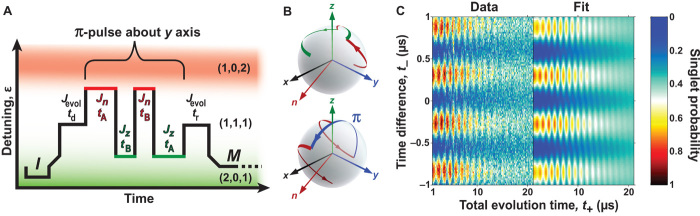
The Y-echo sequence. (**A**) Schematic of the Y-echo sequence. A (2,0,1) singlet is initialized (*I*), and then the detuning is ramped to the (1,1,1) region. Evolution at exchange value *J*_evol_ is interrupted by a series of four pulses alternately around the *n* and *z* axes, which accomplish a composite Y-pulse. Measurement (*M*) is performed as in Fig. 2A. (**B**) Bloch-sphere representation of the Y-pulse. The upper sphere shows the four elements of the composite pulse, with rotations about the *n* axis shown in red and rotations about the *z* axis shown in green. Regardless of the starting location, these four rotations accomplish a π-pulse about the *y* axis, which is depicted as a single blue arc in the lower sphere. The Y-pulse is itself preceded and followed by two evolution periods. In this example, both dephasing and rephasing evolutions amount to rotations by angle π about the *n* axis as shown in red. Any equal rotation during the dephasing and rephasing periods about any combination of *n* or *z* axes would end up at the south pole. (**C**) Singlet probability data and fit for the Y-echo experiment. The difference between dephasing and rephasing times is plotted on the vertical axis (*t*_−_ = *t*_d_ − *t*_r_), and the total evolution time (*t*_+_ = *t*_d_ *+ t*_r_) is plotted on the horizontal axis.

The Y-echo pulse sequence is shown in Fig. 5A. After singlet initialization, exchange oscillations are driven for a dephasing time *t*_d_ at a variable detuning ε at which we intend to study exchange noise. A Y-pulse is then performed, after which oscillations at detuning ε continue for a rephasing time *t*_r._ The composite Y-pulse consists of four pulses of alternating *n* and *z* rotations with angles [θ_A_, θ_B_, θ_B_, θ_A_], where ideally θ_A_ = 145.2219° and θ_B_ = 81.1006°. A depiction of this composite rotation is shown in Fig. 5B. We implement the Y-pulse by pulsing between two detuning values, ε_*z*_ and ε_*n*_, for which exchange is dominated by *J*_*z*_ and *J*_*n*_, respectively. Each detuning pulse is held constant for a duration *t*_A_ or *t*_B_, where *t*_A_/*t*_B_ = θ_A_/θ_B_. To calibrate the detuning values ε_*z*_ and ε_*n*_, we maximize the visibility of the resulting echo, which occurs when *J*_*k*_(ε_*k*_)*t_X_* = θ*_X_* for *k* = *z*,*n* and *X* = A,B.

In the absence of any magnetic field gradients and in the case where the evolution detuning is set for *n* rotations, the Y-echo sequence would record an ensemble-averaged singlet probability of

$$P(t_{+},t_{-})=\frac{3}{8}[1-e^{-{\left(t_{-}/T_{2e}^{*}\right)}^{2}-{\left(t_{+}/T_{2e}\right)}^{2}}\text{cos}(J_{n}t_{-})],$$

where *t*_±_ = *t*_d_ ± *t*_r_. The triple-dot dephasing and decoherence times ($T_{2e}^{*}$ and *T*_2e_) are in this case due to charge noise, unlike the double-dot experiments discussed earlier, which measure double-dot dephasing and decoherence times due to magnetic field gradients ($T_{2m}^{*}$ and *T*_2m_). We model charge noise as a projection onto the detuning voltage ε with a 1/*f* noise spectrum of the form *S*_ε_(*f*) *= A*_e_^2^*/f*, giving *T*_2e_ = [2/ln(2)]^1/2^*ℏ*/[*A|*d*J*/dε*|*]. An average from three-parameter data fits of the central echo (*t*_−_ = 0) over 11 different values of evolution exchange *J*_*n*_ ranging from *J*_*n*_*/h* = 0.4 to 6.8 MHz gives *A*_e_ = 15 ± 2 μV. This level of 1/*f* noise is of comparable order to the noise observed on the direct time-domain current of the charge sensor of this device when it is tuned near a charge transition.

Noisy magnetic field gradients are still present, and these result in additional oscillations and some magnetic dephasing at time scale $T_{2m}^{*}$. A more complicated expression including these effects is derived using the method of ref. (*27*). This derivation appears in the Supplementary Materials. The complete data set from the Y-echo experiment, shown in Fig. 5C for one particular detuning, fits very well to this model including gradient-induced oscillations and using dephasing times $T_{2e}^{*}$ and $T_{2m}^{*}$ comparable to those observed in the Rabi and Ramsey oscillations already discussed. Data and fits for a range of detunings are shown in the Supplementary Materials.

## DISCUSSION

We have used our ability to perform universal control of an all-electrically controlled qubit with composite exchange pulses to measure the levels of magnetic and charge noise in isotopically purified silicon devices. Further exploration of materials and fabrication methods will be necessary because these experiments do little to identify the physical origins of both of these noise sources. However, these noise sources are already found to be small enough to enable the longer pulse sequences required by exchange-only controlled-NOT (*16*, *29*), dynamical decoupling (*26*), and randomized benchmarking. These will bring electrically controlled semiconductor qubits closer to the goal of useful quantum information processing.

## MATERIALS AND METHODS

Measurements were performed in a ^3^He/^4^He dilution refrigerator with a base temperature of 20 mK. The effective electron temperature based on linewidth measurements of dot charge transitions was estimated to be 80 mK. There were three high-frequency electrical cables going to gates P1, P2, and P3 with a bandwidth of 3 GHz. The current of the quantum dot charge sensor underneath the M gate was amplified at room temperature with 10-kHz bandwidth and then digitized with an A/D converter at 1-μs intervals. Because of the bandwidth of the electronics, a 100-μs wait was implemented at the beginning of each measurement segment (see Fig. 2A) before collecting data for another 100 μs. To achieve *T*_1_ times exceeding milliseconds, the tunneling rate between (2,0,1) and (1,0,1) was lowered by changing the DC bias on the T1 gate. As a result, the time needed to initialize a (2,0,1) singlet was about 50 μs, thus making the whole measurement procedure 450 μs long.

**Fig. 6:**
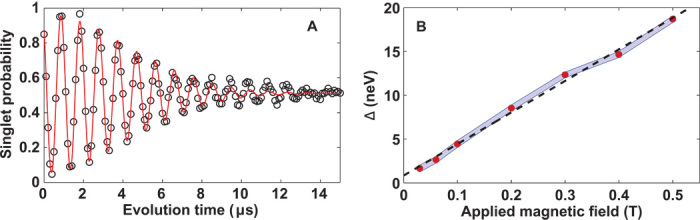
Paramagnetic oscillations. (**A**) Averaged double-dot singlet-triplet oscillations at a magnetic field of 0.1 T. The open circles show the result of 512 averages of single-shot singlet measurements taken over a total of 107 s. The red curve is a best-fit Gaussian-damped cosine with 〈Δ〉/*h* = 1.070 ± 0.001 MHz and $T_{2m}^{*}$ = 6.4 ± 0.3 μs. (**B**) The mean singlet-triplet splitting from averaged double-dot singlet-triplet oscillations as a function of applied magnetic field. Red dots are results of curve-fitting data, as in (A), but with longer averaging times of about 2 hours. The blue band shows the distribution $\sigma_{\Delta }=\hbar \sqrt{2}/T_{2m}^{*}$ about each mean frequency from the same fits, and the dashed black line shows a linear fit with slope 36 ± 1 neV/T.

To measure *T*_1_ with the device tuned as a double dot, we used the intrinsic paramagnetic field gradient for singlet-triplet oscillations. This gradient was observed as a fixed nonzero oscillation frequency 〈Δ〉/*h* in time-averaged double-dot singlet-triplet oscillations; an example is shown in Fig. 6A. By curve-fitting these oscillations and changing the applied in-plane magnetic field, we see in Fig. 6B that 〈Δ〉/*h* is proportional to magnetic field. Presumably, the source of this gradient is also the source of nonnuclear magnetic gradient fluctuations, which we discuss in the Supplementary Materials.

**Fig. 7:**
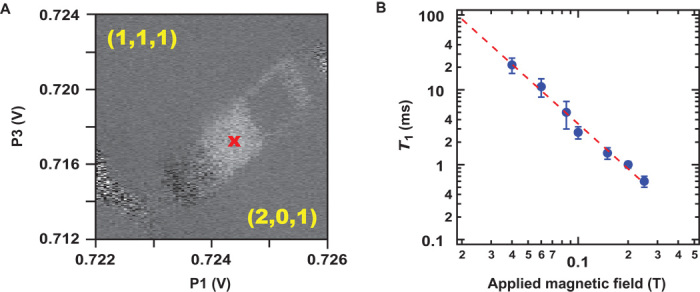
*T*_1_ measurements. (**A**) Plot of current versus the DC bias on P3 and P1 while running a pulse sequence creating triplets utilizing the paramagnetic field. The white square depicts the bias space where Pauli blockade occurs, and the red “x” denotes the location where *T*_1_ measurements were made. (**B**) A plot of *T*_1_ of triplets in the readout window versus external magnetic field. The blue points show the fit values and uncertainty of exponential triplet decay rate as a function of wait time, and the dashed red line is a fit to *T*_1_ ∝ 1/*B*^2^.

The *T*_1_ measurement used the pulse sequence shown in Fig. 2A. During the evolution, triplets were created because of the paramagnetic gradient. To measure triplet relaxation, we vary the wait time in the first measurement segment, with voltages parked in the readout position shown in Fig. 7A, and observe the reduction in triplet population. During *T*_1_ relaxation in the double-dot, strong exchange (*J*_*z*_) between P1 and P2 was present. Fig. 7B shows the dependence of *T*_1_ versus the externally applied in-plane magnetic field *B*; it is found that *T*_1_ ∝ 1/*B*^2^.

The Rabi-Ramsey and Y-echo experiments were performed at external magnetic fields near zero. According to Fig. 7B, *T*_1_ > 100 ms, and thus *T*_1_ relaxation played a negligible role in measurement errors. Measurement error at low field may also occur because of imperfect discrimination of singlets and triplets (evident in Fig. 2B), as well as drifts in measurement current values and voltage bias points. Our measurements of these effects predict a visibility of low-field oscillations in this work to be better than 99.9%. In practice, however, the zero-field visibility found from curve-fitting double-dot paramagnetic oscillations is 98 ± 1%. The reduced visibility may be due to errors in the singlet initialization, which happen twice per single-shot experiment. At the higher magnetic fields used to study noise in the paramagnetic gradient, the visibility worsens as expected because of triplet *T*_1_ decay occurring during the wait time (100 μs) and measure time (100 μs).

## Supplementary Material

http://advances.sciencemag.org/cgi/content/full/1/4/e1500214/DC1

## SUPPLEMENTARY MATERIALS

Supplementary material for this article is available at http://advances.sciencemag.org/cgi/content/full/1/4/e1500214/DC1 Detailed Magnetic Noise Measurements Triple-Dot Data Analysis Fig. S1. Magnetic gradient noise. Fig. S2. Magnetic gradient spin echo. Fig. S3. Fit to sample row of triple-dot Rabi data. Fig. S4. Three-parameter fits to all Y-echo data. Fig. S5. Fit to *t*_−_ = 0 band of Y-echo data.
