# Supplementary material for: Isotopically enhanced triple-quantum-dot qubit
Source: Sci Adv. 2015 May 29;1(4):e1500214. doi: 10.1126/sciadv.1500214 (PMC4640653; doi:10.1126/sciadv.1500214)
Supplement: http://advances.sciencemag.org/cgi/content/full/1/4/e1500214/DC1 [file supp_1_4_e1500214__index.html]

Science Advances | Science Advances

## Supplementary Materials

**This PDF file includes:**

- Detailed Magnetic Noise Measurements
- Triple-Dot Data Analysis
- Fig. S1. Magnetic gradient noise.
- Fig. S2. Magnetic gradient spin echo.
- Fig. S3. Fit to sample row of triple-dot Rabi data.
- Fig. S4. Three-parameter fits to all Y-echo data.
- Fig. S5. Fit to *t*− = 0 band of Y-echo data.

Download PDF

**Files in this Data Supplement:**

- Adobe PDF - 1500214\_SM.pdf
