# Supplementary material for: Isotopically enhanced triple-quantum-dot qubit
Source: Sci Adv. 2015 May 29;1(4):e1500214. doi: 10.1126/sciadv.1500214 (PMC4640653; doi:10.1126/sciadv.1500214)
Supplement: http://advances.sciencemag.org/cgi/content/full/1/4/e1500214/DC1 [file 1500214_SM.pdf]

Science Advances

www.advances.sciencemag.org/cgi/content/full/1/4/e1500214/DC1

Supplementary Materials for

Isotopically enhanced triple-quantum-dot qubit

Kevin Eng, Thaddeus D. Ladd, Aaron Smith, Matthew G. Borselli, Andrey A. Kiselev, Bryan H. Fong, Kevin S. Holabird, Thomas M. Hazard, Biqin Huang, Peter W. Deelman, Ivan Milosavljevic, Adele E. Schmitz, Richard S. Ross, Mark F. Gyure, Andrew T. Hunter

Published 29 May 2015, *Sci. Adv.* **1**, e1500214 (2015)
DOI: 10.1126/sciadv.1500214

This PDF file includes:

Detailed Magnetic Noise Measurements
Triple-Dot Data Analysis
Fig. S1. Magnetic gradient noise.
Fig. S2. Magnetic gradient spin echo.
Fig. S3. Fit to sample row of triple-dot Rabi data.
Fig. S4. Three-parameter fits to all Y-echo data.
Fig. S5. Fit to $t_{-} = 0$ band of Y-echo data.

Detailed Magnetic Noise Measurements:

As indicated in the main text, we see substantial evidence that magnetic gradient noise arises from a non-nuclear source. The first evidence is the paramagnetic gradient discussed in the previous section with evidence shown in Fig. 6 of the main text. This gradient would be extremely difficult to explain with thermally or dynamically polarized ^{29}Si or ^{73}Ge nuclear spins.

Perhaps more telling is the temporal fluctuation in the gradient-induced splitting $\Delta(t)$. This can be deduced by Fourier-transforming and curve-fitting a single-shot of the singlet-triplet oscillations to extract Δ, and observing its direct time-domain fluctuation. An example trace appears in Fig. S1A. The fluctuation has a shorter correlation time than expected for diffusing nuclear magnetization in 800 ppm ^{29}Si. A typical noise spectrum for the measured gradient is shown in Fig. S1B, which appears to follow a $1/f^{1.5}$ trend at high magnetic fields. As a function of magnetic field, a $1/f^{\alpha}$ spectrum is always observed with α varying from 1 to about 1.5 as the field is increased, as shown in Fig. S1C.

We note that for noise with a $1/f^{\alpha}$ characteristic, T_2^* depends on the averaging time. If dephasing is truly due to a finite nuclear ensemble, then one may define T_2^* as the dephasing time after infinite ensemble averaging (or, more practically, sufficient averaging to sample the entire available nuclear hyperfine distribution). However, this averaging time may be prohibitively long, especially for diffuse nuclear ensembles. Moreover, infinite ensemble averaging over a true $1/f^{\alpha}$ noise distribution would lead to vanishing T_2^*. If measuring substantially faster than the time it takes to sample the underlying gradient distribution, measurements of T_2^* will be longer. In Fig. S1D, we show the T_{2m}^* times we extract by averaging only a subset of the total number of ensemble measurements, as a function of the actual laboratory time for that subset. We see that for our $1/f^{\alpha}$ noise source, especially for higher values of α, faster measurements lead to longer effective T_{2m}^* times. One must therefore be cautious when comparing T_{2m}^* numbers measured with finite averaging times. The expected infinite-averaging-time T_{2m}^* due to 800 ppm ^{29}Si (corresponding to about 200 nuclear spins in the 5000 nm^3 effective dot volume) is not grossly different than the 2.5 μs time we observe at low field, but given the slow nuclear diffusion time and finite averaging time, a much longer T_{2m}^* result would likely be observed.

Finally, the $1/f^{\alpha}$ character of magnetic noise is confirmed by measuring spin-echo, in which singlet-triplet oscillations evolve for a dephasing time t_d, a π-pulse is performed via double-dot exchange, and then singlet-triplet oscillations evolve again for a rephasing time t_r (*1*). As a function of the total and difference times, $t_+ = t_d + t_r$ and $t_- = t_d - t_r$, one expects a singlet probability of the form $P_S(t_+, t_-) = \{1 - \cos(\langle\Delta\rangle t_-/\hbar)\exp[-R_\Delta(t_+, t_-)]\}/2$, where $R_x(t_+, t_-)$ is a standard relaxation function for a Hahn-echo-like sequence due to classically

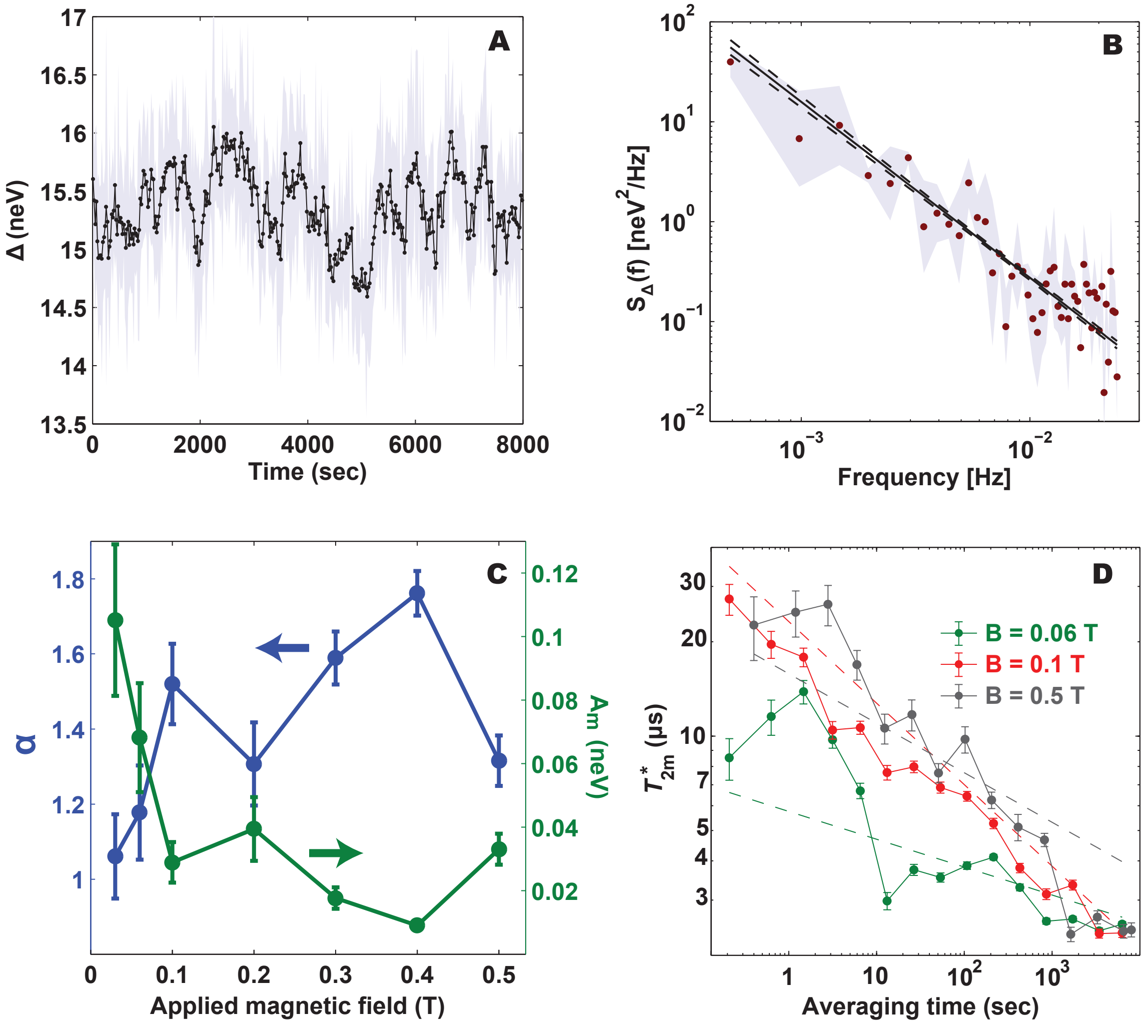

Figure S1: Magnetic Gradient Noise. **A**: Mean spin flip-flop splitting energy $\langle\Delta\rangle$ deduced from singlet-triplet oscillations as a function of laboratory time, as found via curvefitting over averages of 25 single-shot traces. This data is for an applied magnetic field of 0.4 T. The light blue band is the single-σ confidence interval for the fit. **B**: Power spectral density of $\langle\Delta\rangle$. The time trace in **A** is divided into 100 intervals whose power spectra are averaged to give the red dots with standard deviation in light blue. This spectrum is then fit to a power-law; the best fit is the solid black line and the dashed lines represent the single-σ confidence interval. **C**: The results of the power-law fits in **B**. Fit values and standard errors of α (blue points, left axis) and $A_{\rm m}$ (green points, right axis) for $S_\Delta(f) = A_{\rm m}^2 f_0^{\alpha-1}/f^\alpha$ as a function of magnetic field. The reference frequency $f_0 = 1$ Hz is used to keep $A_{\rm m}$ in units of neV. **D**: T^*_{2m} measured via curvefitting as a subset of total averaging time T for three different applied magnetic fields B. The dashed lines are the leading order power-law expectation, $T^*_{2\rm m} \sim (\hbar/A_{\rm m})\sqrt{2(\alpha-1)/(f_0 T)^{\alpha-1}}$, with $A_{\rm m}$ and α given by the values in **C**.

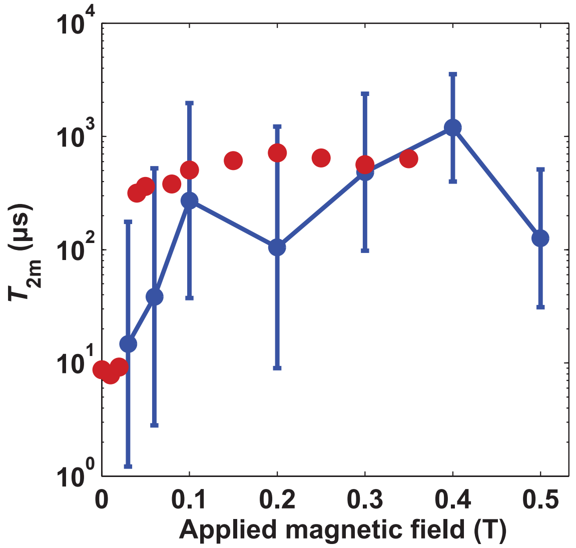

Figure S2: Magnetic Gradient Spin Echo. Magnetic gradient T_{2m} measured via Hahn spin echo (*1*) as a function of magnetic field. The red dots show the $1/e$ decay point of the measured spin-echo decay, and the blue dots show the integral of $A_{\rm m}^2 f_0^{\alpha-1}/f^{\alpha}$, with A and α and their uncertainties given in Fig. S1, to the Hahn echo filter function (Eq. (1), with $t_d = t_r$).

fluctuating parameter x, given by

$$\begin{aligned} &R_x(t_+, t_-) \\ &= \frac{1}{\hbar^2}\int \frac{d\omega}{2\pi} S_x(\omega)\left\{\int_0^{t_d} dt_1 \int_0^{t_1} dt_2 \cos[\omega(t_1-t_2)] + \int_{t_d}^{t_d+t_r} dt_1 \int_{t_d}^{t_1} dt_2 \cos[\omega(t_1-t_2)] \right. \\ &\qquad \left. - \int_{t_d}^{t_d+t_r} dt_1 \int_0^{t_d} dt_2 \cos[\omega(t_1-t_2)]\right\} \\ &= \frac{1}{\hbar^2}\int \frac{d\omega}{\pi\omega^2} S_x(\omega)\{\sin^2(\omega t_d/2) + \sin^2(\omega t_r/2) - 2\sin(\omega t_d/2)\sin(\omega t_r/2)\cos(\omega t_+/2)\}. \end{aligned} \tag{1}$$

The term in the brackets is the filter function for the Hahn echo; in particular, when $t_d = t_r$ we obtain $F(\omega t_+) = 2\sin^2(\omega t_+/4)(1-\cos(\omega t_+/2)) = 4\sin^4(\omega t_+/4) \sim \omega^4$. When integrated against a spectral noise density of the form $S_\Delta(f) = A^2 f_0^{\alpha-1}/f^{\alpha}$, one obtains

$$R_\Delta(t_+, 0) = \frac{A^2}{\hbar^2}(\pi f_0)^{\alpha-1} t^{1+\alpha}(1-2^{\alpha-1})\Gamma(-1-\alpha)\sin\left(\frac{\pi\alpha}{2}\right). \tag{2}$$

Figure S2 shows the measured spin-echo decay time as a function of magnetic field in red, which may be compared to the blue dots showing the expected spin-echo decay time (i.e. the value of

t_+ where $R_\Delta(t_+, 0) = 1$) corresponding to Eq. (2) and the direct time-domain measurements of A and α shown in Fig. S1.

Evident from Figures S1C and S2 is a clear change in the noise character of the fluctuating gradient $\Delta(t)$ in the vicinity of an applied magnetic field of 0.04 T. This field corresponds to where the Zeeman energy of an electron spin equals the thermal energy close to the base temperature of the dilution refrigerator. This is highly suggestive of an electronic source for this gradient noise, although the material origin remains unknown.

Triple-Dot Data Analysis:

In this section, we discuss the models to which we fit our triple-dot data. For both the triple-dot Rabi/Ramsey experiment (Fig. 4) and the Y-echo experiment (Fig. 5), we make a couple of simplifying assumptions. One assumption we make is to treat magnetic gradient fluctuations as quasi-static, zero-mean Gaussian noise. At the magnetic fields close to zero used for these experiments, this approximation fits the data well, especially since most of the data is dominated by exchange noise that renders the effects of dynamic gradient fluctuations difficult to detect.

For our second simplifying assumption, we note that in the Rabi, Ramsey, and Y-echo sequences, we combine short π-pulses with free evolution. In the Rabi and Ramsey cases, there is an initial and final π-pulse about the n-axis; in the Y-echo case, there is a single composite π pulse about the y-axis in the middle of the sequence. For purposes of fitting data with a simple model, we will treat these π pulses as instantaneous, and treat pulse errors only as a reduction of total fringe visibility. In reality these pulses certainly have finite duration and errors correlated to the surrounding evolution, but these have little impact on the shape of the observed oscillations and therefore little impact on extracting physical parameters using the resulting models.

To derive simple analytic models, we use two important types of basis change, both discussed further in Ref. 27. They are expressed as rotations in SU(3), using the notation $U_j(\theta) = \exp(-i\theta\lambda_j/2)$ for the Gell-Mann matrix λ_j. The first important basis change is performed by the rotation $U_2(\phi)$, where $\phi = 120°$. In the logical qubit space, this is a rotation about the y-axis by angle ϕ. This operation is also equivalent, up to simple geometric reflections, to a permutation of the dot labels. We define that exchange among dots 1 and 2 defines rotations about z, and that exchange between 2 and 3 defines rotations about n, but we could reassign these dot assignments and simultaneously perform the $U_2(\phi)$ basis change, taking care to update associated initialization and measurement states. Hence the Hamiltonian terms due to exchange are written

$$J_z \vec{S}_1 \cdot \vec{S}_2 = -\frac{J_z}{2}\left[\lambda_3 + \frac{\lambda_8}{\sqrt{3}}\right], \tag{3}$$

$$J_n \vec{S}_2 \cdot \vec{S}_3 = -\frac{J_n}{2} U_2(\phi)\left[\lambda_3 + \frac{\lambda_8}{\sqrt{3}}\right] U_2^\dagger(\phi), \tag{4}$$

where $\vec{S}_j$ is the spin-vector for the electron in dot j.

The second important basis change uses the observation that if only one exchange interaction is active, then the simplest basis in which to calculate is the 2-dimensional subspace of the double-dot qubit for which exchange acts as a Z-rotation. The transformation between the "triple-dot" and "double-dot" basis is achieved by the unitary $U_7(\beta)$, where $\beta = \pi - \tan^{-1}\sqrt{8}$. In the double-dot basis, say for dots 1 and 2, the magnetic gradient energy between dots 1 and 2, notated $\Delta_{12} = g\mu_{\mathrm{B}}(B_1 - B_2)$, causes x-axis rotations, while the difference between the average magnetic energy on dots 1 and 2 and the field of the third dot, notated $\Delta_{\overline{12}} = g\mu_{\mathrm{B}}[B_3 - (B_1 + B_2)/2]$, only causes a phase difference for the third, non-qubit state. Hence the Hamiltonian term due to magnetic noise is written

$$H_{\mathrm{B}}(\pm[B_1, B_2, B_3]) = \pm U_7(\beta)\left[\frac{\Delta_{12}}{2}\lambda_1 + \frac{\Delta_{\overline{12}}}{\sqrt{3}}\lambda_8\right]U_7^\dagger(\beta). \quad (5)$$

The $\pm$ reflects the fact that the two SU(3) subspaces of the triple-dot system (corresponding to the total spin projection) rotate in opposite orientations with respect to magnetic field gradients. However, the two subspaces perform equivalently on average when treating an ensemble of noisy field gradients with zero mean. The $U_7(\beta)$ transformation block diagonalizes the Hamiltonian into an SU(2) and a U(1) block, which are trivial to exponentiate. We notate the SU(2) exponential as

$$\begin{aligned} V(J, \pm[B_j, B_k, B_\ell], t) &= U_7^\dagger(\beta)\exp\left[-i\frac{Jt}{\hbar}\vec{S}_j\cdot\vec{S}_k - iH_{\mathrm{B}}(\pm[B_j, B_k, B_\ell])\frac{t}{\hbar}\right]U_7(\beta) \\ &= \exp\left\{i\frac{Jt}{2\hbar}\left[\lambda_3 + \frac{\lambda_8}{\sqrt{3}}\right] \mp i\frac{t}{\hbar}\left[\frac{\Delta_{jk}}{2}\lambda_1 + \frac{\Delta_{\overline{jk}}}{\sqrt{3}}\lambda_8\right]\right\}. \end{aligned} \quad (6)$$

In what follows, we use basis changes and dot permutations to cast evolution into this easily calculated exponential.

Triple-dot Rabi/Ramsey: The triple-dot Rabi/Ramsey experiment demonstrates the basic operation of the qubit. However, it shows more complicated relaxation phenomena than analogous double-dot experiments. We are nonetheless able to fit these phenomena to a relatively simple model that we now discuss.

The time-dependent evolution in the triple-dot Rabi experiment is described by rotations about the n-axis at exchange rate J_n as well as evolution due to magnetic field gradients. This is exactly the case treated in Ref. 27. We simplify by factoring out the basis change which switches to the double-dot qubit for dots 2 and 3, and permuting the magnetic fields corresponding to the $U_2(\phi)$ transformation. Then the time-dependent evolution during n-rotations given (2,3) exchange J_n is

$$U_n(t) = U_2(\phi)U_7^\dagger(\beta)V(J_n, \pm[B_2, B_3, B_1], t)U_7(\beta)U_2^\dagger(\phi). \quad (7)$$

In the Ramsey case, the time-dependent evolution of the sequence is governed by rotation about the z-axis, so the evolution during the z-rotations given (1,2) exchange J_z is simply

$$U_z(t) = U_7(\beta)V(J_z, [B_1, B_2, B_3], t)U_7^\dagger(\beta). \quad (8)$$

The initial and final π-pulses about the n-axis, which are modeled as perfect and notated N, perform a SWAP$_{23}$ operation, i.e. a swap between spins 2 and 3. This operation therefore adds a SWAP$_{23}$ in the case of Rabi. For Ramsey, we reinterpret this pulse as its equivalent 120° rotation about the y-axis accompanied by a SWAP$_{12}$, giving

$$NU_n(t)N = e^{-i\phi}U_2(\phi)U_7^\dagger(\beta)V(J_n, \pm[B_3, B_2, B_1], t)U_7(\beta)U_2^\dagger(\phi), \tag{9}$$

$$NU_z(t)N = e^{-i\phi}U_2^\dagger(\phi)U_7^\dagger(\beta)V(J_z, \pm[B_2, B_1, B_3], t)U_7(\beta)U_2(\phi). \tag{10}$$

These expressions are very similar, and if we assume that the magnetic field on each dot fluctuates independently with zero mean and with the same variance, the resulting averaged evolution will be the same in all cases. Hence we provide a single expression, referring generally to exchange rate J and to $\sigma_B = (g\mu_{\rm B})^2\langle B_j^2\rangle$. With singlet initialization, the ensemble-averaged singlet probability after evolution time t is

$$P_S(t) = \frac{1}{2} - \frac{\hbar^2}{8\pi\sqrt{2}}\int\int\frac{dy}{\sigma_J}\frac{db}{\sigma_B}e^{-b^2/(4\sigma_B^2)-(y-J)^2/(2\sigma_J^2)}\times \left[\frac{(yt)^2}{8}\text{sinc}^2\left(\frac{\sqrt{b^2+y^2}}{2}t\right) - e^{-3(\sigma_B t)^2/4}\text{Re}\{(1+e^{iyt})e^{i(\sqrt{b^2+y^2}-y)t/2}\}\right]. \tag{11}$$

This is the same expression as Eqs. (9-11) of Ref. 27 except that we have added an integration of J about a quasi-static Gaussian distribution with variance σ_J. A notable feature is the presence of three distinct timescales at high J values, which are visible in the Rabi-Ramsey data. First, there is a decay of oscillations due to magnetic gradient-noise terms that commute with exchange. Second, there is a bias-dependent decay of oscillations due to charge noise. Finally, after charge noise dephases Rabi oscillations to an incoherent singlet population of 1/2, magnetic noise eventually mixes the entire SU(3) subspace, leading to a decay of this incoherent population to 1/3.

Deviations from this model will occur at J values lower than a few MHz, where the assumption of only a single active exchange begins to lose validity. In this region, our model calculates the expected shape by diagonalizing the 3×3 Hamiltonian with both J_z and J_n terms present. The diagonalization is numerically averaged about the Gaussian noise distributions for all exchange values and magnetic terms.

We fit running averages of each row of the Rabi/Ramsey data of Fig. 4 of the main text to this model, including scale parameters to incorporate state preparation, measurement, and π-pulse errors. An example such fit is shown in Fig. S3. The resulting fit values of J_n and J_z are then each fit to a 4-parameter model.

The model we employ for J_n and J_z follows the canonical Hubbard-model result $J = \sqrt{4t_c^2 - \alpha_{\rm L}^2(\epsilon-k_1)^2/4} - \alpha_{\rm L}|\epsilon-k_1|/2$. (We presume each side of the triple-dot (1,1,1) region, and therefore J_z and J_n, each have a different t_c and fitting parameters k_j, but we will discuss both at once here.) In this equation t_c is the tunnel-coupling and k_1 is the first fitting parameter, corresponding to the voltage bias which provides double-dot tunnel resonance. The lever-arm

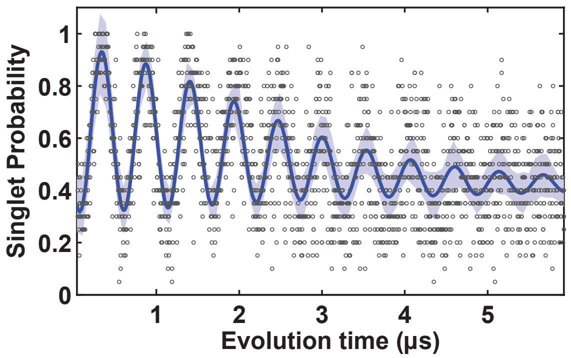

Figure S3: Fit to sample row of triple-dot Rabi data. The raw data points (which average over 20 singles shots) are shown as grey dots; these correspond to detuning voltage -14.25 mV of Fig. 4. The blue curve shows the best fit to $a + bP_S(t + t_0)$, where $P_S(t)$ is given by Eq. (11) and the fitting parameters for this particular detuning with standard error are $a = 0.11 \pm 0.02, b = 0.84 \pm 0.02, t_0 = 0.19$ μsec, $J/h = 1.875$ MHz $\pm$ 5 kHz, $\sigma_B/h = 63 \pm 5$ kHz, and $\sigma_J/h = 41 \pm 5$ kHz. The light blue shaded region is the single-σ confidence interval for the fit corresponding to the given standard errors.

$\alpha_{\rm L}$ we estimate as 0.1 meV/mV, although from a fitting standpoint this parameter may be considered to be absorbed into t_c over the active fitting range. However, we find that this model with a constant t_c is insufficient to explain the dependence of J with detuning voltage ϵ. Since the detuning bias is applied to P1 and P3 gates rather than to P1/P2 or P2/P3 pairs, and possibly because of asymmetries in the device, t_c appears to be a function of ϵ.

Therefore, we use a more elaborate physical model for the tunnel coupling. This model is motivated by three-dimensional self-consistent device-level simulations. These simulations indicate that the plunger and exchange gates collectively form a deep potential trough with 3 to 4 meV spatial quantization energy in the quantum well along the gate row. The quantum dot states are situated inside this trough with a notably weaker quantization of about 2 meV or less, suggesting that the electron character in the trough is approximately one-dimensional. After separating fast and slow electron motion perpendicular and along the trough, and averaging over the fast motion, we end with an effectively one-dimensional potential profile. An application of the Wentzel-Kramers-Brillouin (WKB) approximation is then appropriate to quantify electron interdot tunnel coupling t_c through smooth and shallow barriers separating individual P1, P2, and P3 states. In this approximation, t_c is primarily determined by the exponentiated integral $\mathcal{S}$ of the electron momentum over the classically forbidden barrier region between two turning points, with a prefactor, treated here as another constant fitting parameter k_2. In the WKB approximation, the prefactor is defined by the degree of quantization of dot states on both sides of the barrier. As the height and curvature of the barriers is controlled by the gate biases, it is appropriate to expand the barrier momentum integral as a series in the detuning voltage ϵ.

Keeping only the linear approximation, $\mathcal{S} = k_3 + k_4\epsilon$, where k_3, k_4 are again fitting parameters related to the barrier shape and its dependence on ϵ.

The resulting four-parameter-per-exchange-side model for J follows the experimentally observed oscillation frequency extremely well. Using this function, shown in the rightmost panel of Fig. 4 of the main text, we find that the entire data set fits well to a model with a constant σ_B/h of 63 ± 5 kHz and $\sigma_J = |dJ/d\epsilon|\sigma_\epsilon$ with $\sigma_\epsilon = 70 \pm 5$ μV.

Y-echo: The Y-echo experiment is useful both for extracting charge noise dynamics and for showing the ability to achieve useful universal control in the triple-dot. In the following, we develop a rather complex but still analytic model for the Y-echo in the presence of magnetic gradients, and we see that the observed data matches this model quite well.

In analyzing the Y-echo experiment, we make an additional simplifying assumption. We note that the Δ_{12} terms during free evolution contribute errors of order $(\Delta_{12}/J_n)^2$, which we can ignore under the assumption $\Delta_{12} \ll J_n$. In the Rabi/Ramsey experiment, these terms are mostly indiscernible due to charge noise damping. The approximation is of use because the expression will be more complicated, as we now must consider dynamic noise, treated via the time-varying fluctuation Hamiltonian

$$H_1(t) = -\frac{\delta(t)}{2}\left[U_2(\phi)\lambda_3 U_2^\dagger(\phi) + \frac{\lambda_8}{\sqrt{3}}\right] = -\frac{\delta(t)}{2}U_2(\phi)U_7^\dagger(\beta)\left[\lambda_3 + \frac{\lambda_8}{\sqrt{3}}\right]U_7(\beta)U_2^\dagger(\phi). \quad (12)$$

We will abbreviate the basis change accomplished by $U_2(\phi)U_7^\dagger(\beta)$ with a prime; for example, $H_1'(t) = U_7(\beta)U_2^\dagger(\phi)H_1(t)U_2(\phi)U_7^\dagger(\beta)$.

We will employ time-dependent perturbation theory in the interaction picture. Our unperturbed evolution is approximated to include only J_n rotations, the magnetic gradient evolution which commutes with J_n rotations, and the perfect composite Y pulse at time $t_d = (t_+ + t_-)/2$. Therefore, for times within the dephasing interval,

$$U_0'(t \leq t_d) = \exp\left[-i\frac{J_n}{2}\lambda_3 + i\frac{\Delta_{\overline{12}} - J_n/2}{\sqrt{3}}\lambda_8\right] \quad (13)$$

and the fluctuation Hamiltonian in the interaction picture, $\tilde{H}_1'(t < t_d)$, is equivalent to $H_1'(t)$. Treated ideally (with no error and instantaneous), the composite Y pulse provides the unitary

$$Y = \lambda_2 + e^{i\eta}\left|Q\right\rangle\!\left\langle Q\right|, \quad (14)$$

i.e. it rotates the qubit subspace by π about λ_2 and applies a relative phase η to the leaked state $|Q\rangle$. The angle η is 3.9068 for the 4-pulse sequence beginning with rotation sequence $n - z - n - z$ and angles $2.525, 1.415, 1.415, 2.525$. (All angles here are in radians). In the reference frame in which $H_1(t)$ is diagonal, the composite Y-pulse unitary is

$$Y' = U_7(\beta)U_2^\dagger(\phi)YU_2(\phi)U_7^\dagger(\beta) = \frac{e^{i\eta}}{3} + \frac{1}{\sqrt{3}}\lambda_2 - \frac{e^{i\eta}}{3}\lambda_3 + \sqrt{\frac{2}{3}}\lambda_5 - \frac{\sqrt{2}e^{i\eta}}{3}\lambda_6. \quad (15)$$

We then evolve to total time $t_d + t_r = t_+$, so that for $t > t_d$,

$$U_0'(t) = U_0'(t - t_d) Y' U_0'(t_d). \tag{16}$$

The interaction picture hamiltonian during the rephasing interval is then

$$\tilde{H}_1'(t > t_d) \approx -\tilde{H}_1'(t < t_d) - \frac{\delta(t)}{3}\left[\lambda_3 + \sqrt{2}\cos[\xi(t - t_d)]\lambda_6 - \sqrt{2}\sin[\xi(t - t_d)]\lambda_7\right], \tag{17}$$

where terms of order $(\Delta_{23}/J_n)^2$ have been omitted and

$$\hbar\xi = J_n(\sec\theta - 1)/2 + \Delta_{\overline{23}} \approx \Delta_{23}^2/(4J_n) + \Delta_{\overline{23}}. \tag{18}$$

For a perfect echo, we would desire $\tilde{H}_1'(t > t_d) = -\tilde{H}_1'(t < t_d)$, but we see an extra term in Eq. (17). When $\Delta = 0, \xi = 0$, and in the qubit basis the extra term looks like $U_2(\phi)U_7^\dagger(\beta)(\lambda_3 + \sqrt{2}\lambda_6)U_7(\beta)U_2^\dagger(\phi) = \sqrt{3}\lambda_8$, corresponding only to a phase on the leakage space. Corrections in the measured qubit space due to this term are of order $\Delta_{\overline{23}}\delta^2(t)$, which we neglect here under the continued assumption of small gradients. Neglecting this term substantially simplifies our expression, since our simple Hahn-echo structure gives

$$-\frac{1}{\hbar^2}\int_0^{t_+} dt_1 \int_0^{t_1} dt_2 \langle[\tilde{H}_1'(t_1), [\tilde{H}_1'(t_2), \lambda_j]]\rangle = 0, \qquad j = 3, 8 \tag{19}$$

$$-\frac{1}{\hbar^2}\int_0^{t_+} dt_1 \int_0^{t_1} dt_2 \langle[\tilde{H}_1'(t_1), [\tilde{H}_1'(t_2), \lambda^\pm]]\rangle = -R_\delta(t_+, t_-)\lambda^\pm \tag{20}$$

for $\lambda^\pm = \lambda_1 \pm i\lambda_2$. Here, $\langle\cdot\rangle$ refers to an ensemble average over the random noise variable $\delta(t)$, and $R_\delta(t_+, t_s)$ is given by Eq. (1). Under standard assumptions for cumulant expansions, Eqs. (19) and (20) define a time-dependent linear super-operator $\mathcal{L}(t)[\lambda_j]$, under which $\mathcal{L}(t)[\lambda_j] = \lambda_j$ for $j = 3, 8$ and $\mathcal{L}(t)[\lambda^\pm] = \lambda^\pm \exp[-R_\delta(t_+, t_-)]$.

This simple treatment of dynamic noise is superimposed over more complex oscillations of our unperturbed evolution, governed by unitary $U_0(t)$, over which we will average as we did for the Rabi/Ramsey case above. We initialize and measure the singlet $|0\rangle\langle 0|$, with final singlet probability

$$P_S = \left\langle \mathrm{Tr}\left\{U_0^\dagger\,|0\rangle\langle 0|\,U_0\mathcal{L}(t)[|0\rangle\langle 0|]\right\}\right\rangle, \tag{21}$$

where $\langle\cdot\rangle$ here refers to averaging over quasistatic distributions in J and $\Delta_{\overline{12}}$. We write the singlet in the primed frame; the singlet is unaffected by $U_7(\beta)$ but applying $U_2^\dagger(\phi)$ gives

$$|0\rangle\langle 0|' = U_2^\dagger(\phi)\,|0\rangle\langle 0|\,U_2(\phi) = \frac{1}{3} - \frac{\sin\phi}{2}\left[\lambda^+ + \lambda^-\right] + \frac{\cos\phi}{2}\lambda_3 + \frac{1}{2\sqrt{3}}\lambda_8. \tag{22}$$

Hence, by employing Eq. (22) in Eq. (21), we arrive at nine terms in our expression for P_S, three of them including the echo decay function $\exp[-R_\delta(t_+, t_-)]$. Other terms contain oscillations

which dephase when we average. For example, Tr $\left\{U_0'^\dagger(t)\lambda^\pm U_0'(t)\mathcal{L}(t)[\lambda_8]\right\}$ is non-zero and contains oscillations with respect to J_n, but does not include the relaxation function $R_\delta(t_+, t_-)$.

The final result is

$$P_S(t_+, t_-) = \frac{1}{24}\left\{10 - (1+4G)(1+C) + 4F_-(1-C) - B\left[1 - \frac{\sqrt{3}}{2}\left(K_+ - K_- e^{-R_\delta(t_+,t_-)}\right)\right]\right\}, \quad (23)$$

where

$$\begin{aligned} C &= e^{-(\sigma_J t_-/\hbar)^2/2 - R_\delta(t_+,t_-)}\cos(\bar{J}_n t_-/\hbar) \\ K_\pm &= e^{-[\sigma_J(t_- \pm t_+)/\hbar]^2/8}\sin[\eta - \bar{J}_n(t_+ \pm t_-)/2\hbar] \\ F_\pm &= e^{-(\sigma_B t_\pm/\hbar)^2} \\ G &= \frac{1}{2}\left(e^{-[\sigma_B(t_+ - t_-)/\hbar)^2/4} + e^{-[\sigma_B(t_+ + t_-)/\hbar)^2/4}\right) \\ B &= \frac{4}{3}\left(1 - F_+ + 2F_- - 2G\right). \end{aligned}$$

To help make sense of this expression, note that at $t_+ = 0$, $C = F_\pm = G = 1$ and $B = K_\pm = 0$, so $P_S \to 0$. This is because a perfect Y pulse makes the final singlet probability zero. At very high t_+, $C = F_\pm = G = K_\pm = 0$ and $B = 4/3$, so the saturation singlet probability is 23/72, or 32%. Note that there are no oscillations in t_+, which is the point of the Y-echo in contrast to simpler triple-dot exchange echo experiments. Finally, if there is no gradient whatsoever, so that $\sigma_\Delta = 0$, then $F_\pm = G = 1$ and $B = 0$ at all times, reducing the expression to

$$\lim_{\sigma_\Delta \to 0} P_S(t_+, 0) = \frac{3}{8}[1 - e^{-R_\delta(t_+,0)}], \quad (24)$$

the ideal simple echo case.

For the relaxation function $R_\delta(t_+, t_-)$, we consider the $1/f$ noise spectral density

$$S_\delta(f) = \left|\frac{dJ}{d\epsilon}\right|^2 A_\mathrm{e}^2 \begin{cases} 2f_L/(f^2 + f_L^2) & 0 \le f < f_L \\ 1/f & f_L \le f < \infty \end{cases} \quad (25)$$

for which we obtain

$$\lim_{f_L \to 0} R_\delta(t_+, t_-) = \left|\frac{dJ}{d\epsilon}\right|^2 \frac{A_\mathrm{e}^2}{4\hbar^2}\left\{t_-^2\left[3 - 2\gamma + \pi - \ln[(t_+^2 - t_-^2)(\pi f_L)^2]\right] + t_+^2 \ln\left(\frac{4}{1 - t_-^2/t_+^2}\right) + t_+ t_- \ln\left(\frac{1 - t_-/t_+}{1 + t_-/t_+}\right)\right\}, \quad (26)$$

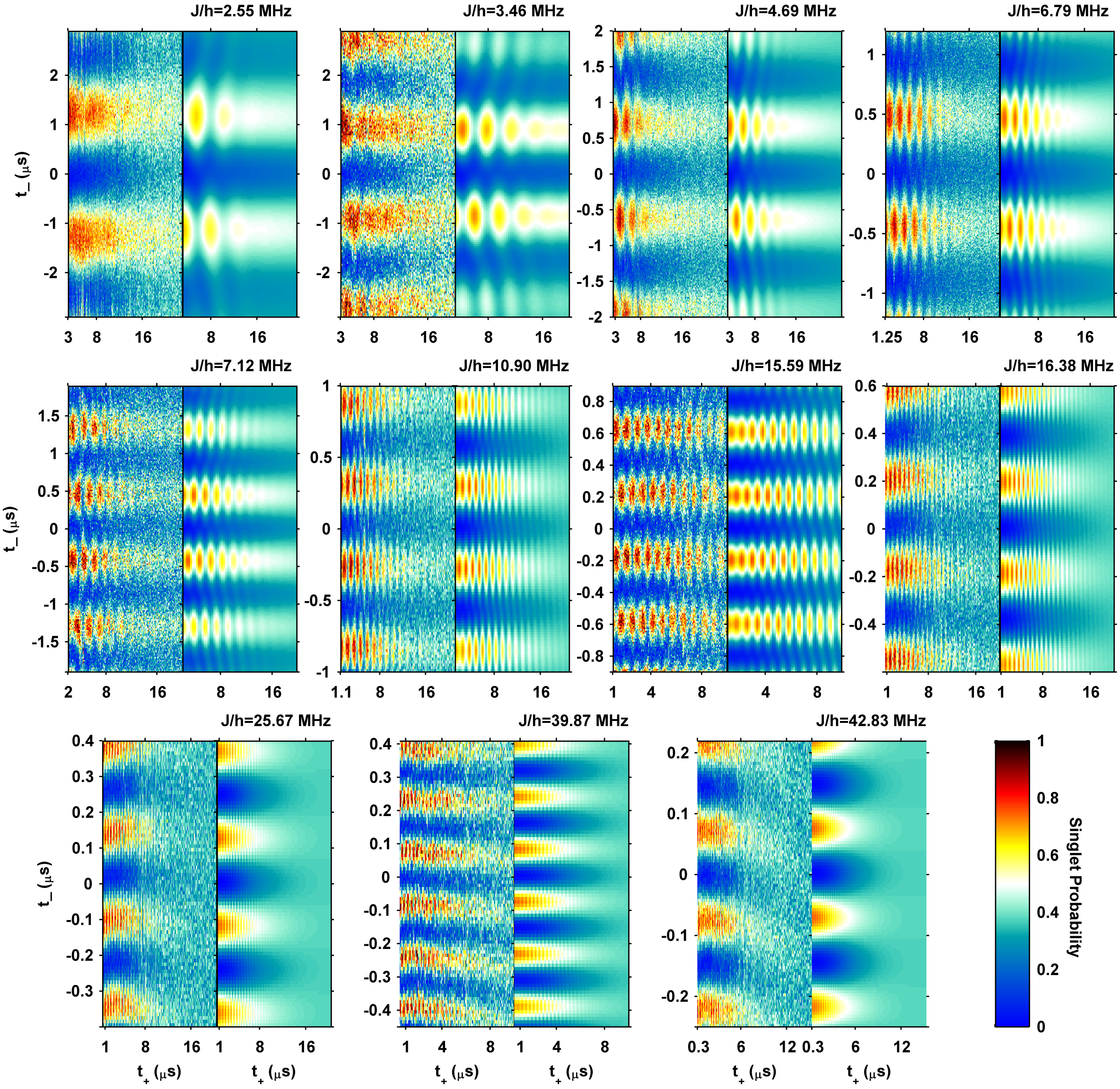

Figure S4: Three-parameter fits to all Y-echo data. In all panels, data is shown on the left and the corresponding fit on the right. The detuning is swept across the data sets, corresponding to changing the J_n value shown to the upper-right of each panel; this value is derived from the fit. The fit function shown is $P_S(t_+, t_-)$ as given by Eq. (23). The second set in the middle row is the same as that in Fig. 5C of the main text.

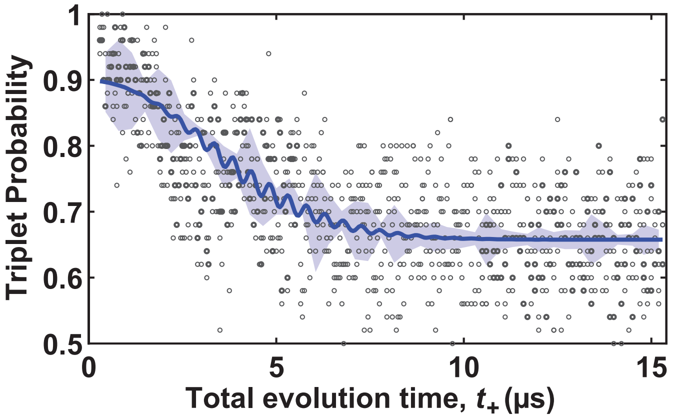

Figure S5: Fit to $t_-= 0$ band of Y-echo data. The raw data points (which are averages over 100 single shots) are shown as grey dots. The blue curve shows the best fit to $a + b(1 - P_S(t_+, 0))$, where $P_S(t_+, 0)$ is given by Eq. (23) using $\sigma_B/h =$ 63 kHz. The fitting parameters for this particular detuning bias with standard error are $a = 0.256 \pm 0.002, b = 0.639 \pm 0.003, J/h = 6.82 \pm 0.02$ MHz, $\sigma_J/h =$ 0.18 ± 0.04 MHz, and $A_\text{e}|dJ/d\epsilon| = 54.5 \pm 0.5$ kHz. The light blue shaded region is the confidence interval for the fit corresponding to the given standard errors.

where γ is Euler's constant. Note that for the perfect echo condition, $t_- = 0$, this expression reduces to simple Gaussian decay:

$$\lim_{f_L \to 0} R_\delta(t_+, 0) = \left|\frac{dJ}{d\epsilon}\right|^2 \frac{A_\text{e}^2 \ln 4}{4\hbar^2} t_+^2. \tag{27}$$

For $t_- \neq 0$, the presence of the factor scaling logarithmically with the low-frequency cut-off f_L is a critical part of the model. This term appears in conjunction with our quasi-static averaging over unfocussed exchange oscillations with variance σ_J^2. In principle, these terms are "double-counted" in our model. Hence we combine these dephasing terms and treat σ_J as a free parameter in our fit, along with A_e. The magnetic field variance σ_B^2 and $|dJ/d\epsilon|$ we take from the Rabi/Ramsey fit, and we can easily find J for each Y-echo data set by simply fitting fringe locations in the t_- direction.

Despite the numerous approximations used to arrive at Eq. (23), this model fits excellently to the many features seen in the data for a wide range of J_n values. Figure S4 shows 11 fits to Y-echo data sets for 11 different values of J. The value $\sigma_B/h = 63$ kHz is used throughout, and no scaling is used. The only fitting parameters are J, σ_J, and A_e. Independent Rabi measurements of J and $dJ/d\epsilon$ are also made, and found to be consistent with the fit results. These whole data set fits show that the model of Eq. (23) captures all of the systematic features of the data. The values of σ_J which result are comparable to those in the Rabi/Ramsey data.

These whole data set fits, however, are poor for gauging the uncertainty of the parameter to be measured, A_e. To be more quantitative, we examine the central lobe of the echo ($t_- = 0$),

whose decay gives the best measure of A_{e} from this data set. Figure S5 shows an example fit to an averaged band around $t_{-} = 0$ for the $J/h = 6.79$ MHz data set shown in Fig. S4. For this particular detuning voltage, $|dJ/d\epsilon|/h = 4.49 \pm 0.01$ MHz/mV. The fit to the central lobe, after dividing the found relaxation time by $|dJ/d\epsilon|$, gives $A_{\mathrm{e}} = 12.1 \pm 0.1$ μV. Here, the uncertainty is that of this particular fit, which underestimates the variance from data set to data set. Across all data sets, we find the estimate of A_{e} increases at lower J values, although we expect A_{e} to be approximately independent of J. The low-J values are less reliable; we find that the uncertainty of the fit also increases as J is reduced. These phenomena occur because as $|dJ/d\epsilon|$ decreases, the absolute charge noise decreases, and magnetic noise dominates; as a result the reliability of the fit decreases. We therefore estimate A_{e} and our uncertainty of it by taking a mean of the central-lobe fit result for the 11 values of J studied, weighted by the inverse variance for each fit. Doing so gives the result $A_{\mathrm{e}} = 15 \pm 2$ μV indicated in the main text.
